# Reconstruction of 100-year dynamics in *Daphnia* spawning activity revealed by sedimentary DNA

**DOI:** 10.1038/s41598-021-03899-0

**Published:** 2022-02-02

**Authors:** Narumi Tsugeki, Kai Nakane, Hideyuki Doi, Natsuki Ochi, Michinobu Kuwae

**Affiliations:** 1 Faculty of Law, Matsuyama University, Matsuyama, 790-8578 Ehime Japan; 2 Center for Marine Environmental Studies, Ehime University, Matsuyama, 790-8577 Ehime Japan; 3 Graduate School of Simulation Studies, University of Hyogo, Kobe, 650-0047 Hyogo Japan

**Keywords:** Ecological genetics, Freshwater ecology, Microbial ecology, Molecular ecology, Palaeoecology, Population dynamics, Restoration ecology, Ecology, Microbiology, Molecular biology, Ecology, Environmental sciences, Limnology

## Abstract

Environmental DNA (eDNA) is currently developing as a powerful tool for assessing aquatic species dynamics. However, its utility as an assessment tool for quantification remain under debate as the sources of eDNA for different species is not always known. Therefore, accumulating information about eDNA sources from different species is urgently required. The objective of our study was to evaluate whether sedimentary DNA targeting two *Daphnia* species, *D. galeata* and *D. pulicaria*, could track *Daphnia* population dynamics and resting egg production. Applying a quantitative PCR targeting the mitochondrial 12S rRNA gene on sediment cores collected in Lake Biwa, Japan, we compared sedimentary DNA concentration of *Daphnia* with the abundance of remains and ephippia, reflecting their abundance and resting egg production, respectively. We found that the sedimentary DNA concentrations of *Daphnia* for the past century were inconsistent with their population abundance. However, the concentration was highly correlated with the resting egg production. Our results provide evidence that ephippia with resting eggs, released during spawning activities, was a significant source of *Daphnia* DNA archived in sediments. Our work provides critical insights for using sedimentary DNA as a monitoring tool for egg production dating back 100 years.

## Introduction

Freshwater ecosystems are among the most at-risk habitats in terms of anthropogenic impact and global and local species loss^1–6^. However, our understanding of these impacts and their role in accelerating habitat disturbance remains limited^7–9^. Environmental DNA (eDNA) is a powerful tool for assessing the loss of biodiversity^10^ and species distributions in aquatic ecosystems^11^. The use of eDNA to estimate population abundance in aquatic systems has the potential to be highly effective as some studies found significant and positive relationships between eDNA concentration and abundance of fish^12–14^. Also for zooplankton read counts from eDNA corresponded to estimated biomass under certain conditions^15^. However, considerable variation exists in the relationship between eDNA and abundance of species in natural systems, which can differ according to the marker used as well as the species under investigation. Consequently, the use of eDNA as a quantifying tool of natural populations remains under debate^11^. Recent studies have also shown that eDNA were highly released during spawning periods^16–18^. Therefore, there is an urgent need for comparisons between eDNA concentrations and species abundance as well as reproductive and spawning activities, such as egg production. However, it is difficult to obtain such data without exhaustive sampling, and to our knowledge, there are limited studies that have made such comparisons^19,20^.

Sedimentary DNA techniques could prove effective for assessing the relative importance of potential sources of eDNA released at different life stages, such as reproduction and spawning activities. Aquatic sediments are generally characterized by relatively high eDNA concentrations compared to what is observed in water columns for fish^21,22^, though little is known on other taxa^19^. Some paleolimnological studies have investigated the congruence between sedimentary DNA and morphological techniques to track community composition of plankton communities^20,23^. However, these studies focused on the community composition of different taxa and not on the comparisons between DNA concentrations and species abundances or egg production.

It is well known that numerous aquatic microorganisms such as plankton have a resting stage, which allows them to withstand stressful environmental conditions^24^. *Daphnia* is a ubiquitous herbivorous freshwater organism that thrives in aquatic systems by producing substantial offspring and switching to resting egg production under unfavorable conditions^25,26^ (Supplementary Fig. S1). These small planktonic crustaceans have been preserved in sediments for centuries^24,27^. Paleolimnological studies have shown that the morphological remains, such as post-abdominal claws and ephippia, archived in lake sediments can be used to reconstruct the resting egg production and population abundance of *Daphnia* over centuries^28,29^. With the aim of gaining a better understanding of the biological responses of *Daphnia* to anthropogenic environmental change^30,31^, recent research studies have increasingly focused on determining the genetic structure of *Daphnia* populations related to long-term environmental change^32,33^. However, there is still a significant information deficit on the comparison of long-term changes in the sedimentary DNA of *Daphnia* species and their abundance and resting egg production.

A previous study on sediment in Lake Biwa, Japan (Supplementary Fig. S2) described temporal changes in the *Daphnia* population abundance and resting egg production during the twentieth century^29^. This reconstruction of population abundance was consistent with existing time-series zooplankton monitoring data^34^. Moreover, for the past several decades, Lake Biwa sediment has had a continuous record with a high time resolution of two years per 1-cm layer sample^35^. These observations suggest that *Daphnia* in Lake Biwa is a suitable for comparing temporal changes in sedimentary DNA and its abundance and resting egg production.

**Figure 1:**
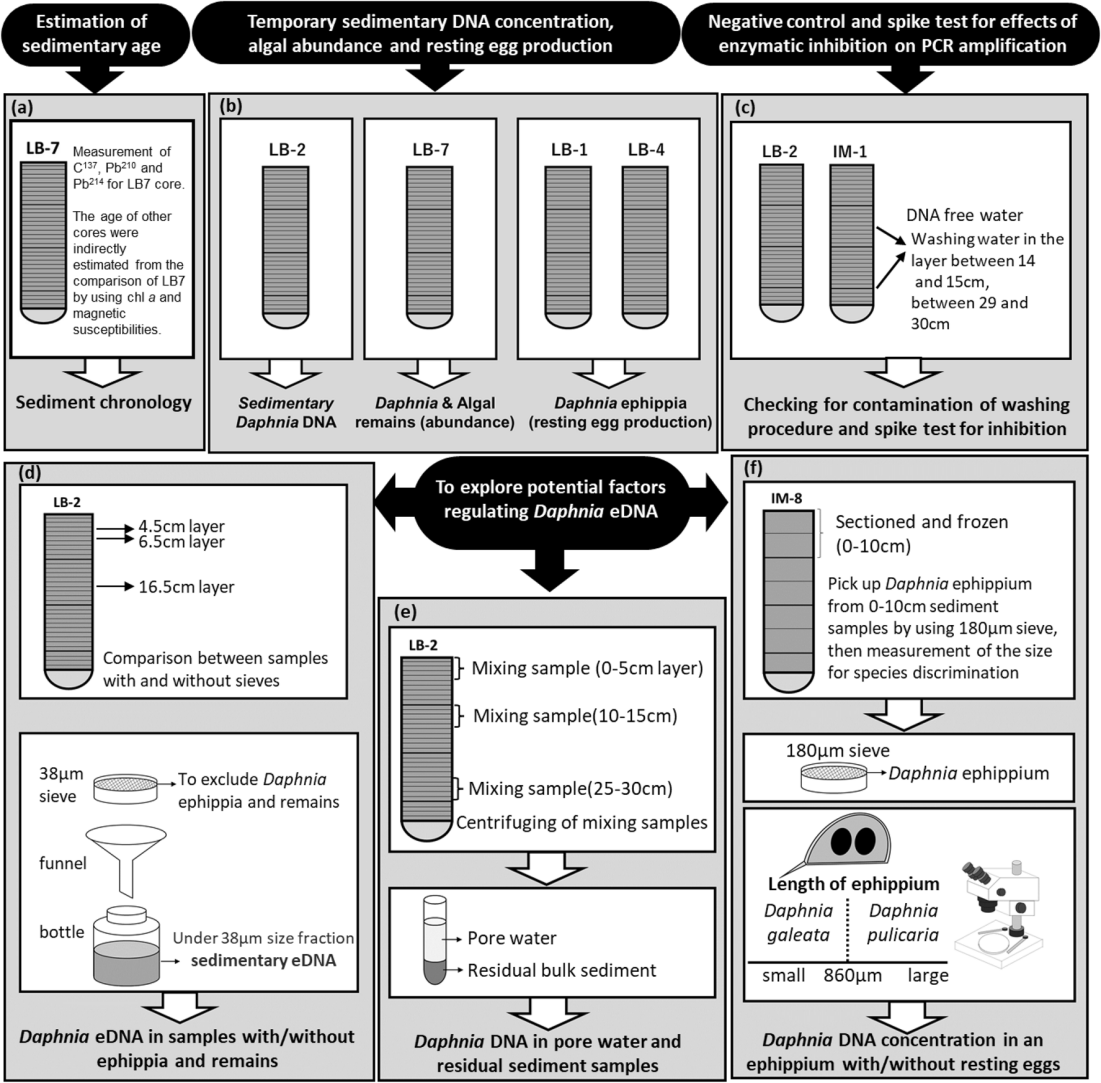
Research and experimental design of the study. (**a**) Sediment geochronology; (**b**) Reconstruction of temporal variation in sedimentary *Daphnia* DNA concentrations, remain abundance and resting egg production as well as algal remain abundance and chlorophyll pigment concentration; (**c**) Contamination and spike test for PCR inhibition; (**d**) Evaluating potential sources of sedimentary *Daphnia* DNA between sample with and without ephippia; (**e**) Potential factors of sedimentary *Daphnia* DNA between pore water and its residual sediment sample; (**f**) Measurement of *Daphnia* DNA per ephippia with and without resting eggs. The named LB cores (namely LB1, LB2, LB4, and LB7) were collected in August 2017, whereas those IM cores (namely IM1 and IM8) were collected in August 2019 (details are described in the section on sampling site and sediment collection of methods).

Here, we explored sedimentary DNA spanning 100 years up to the present day in two *Daphnia* species, *Daphnia galeata* and *Daphnia pulicaria*, extracted from sediment cores from Lake Biwa to determine whether *Daphnia* sedimentary DNA could be used as an indicator to reconstruct the past population abundance and resting egg production, which were reconstructed from their remains and ephippia, respectively, preserved in sediment cores (Fig. 1b). We applied quantitative polymerase chain reaction (qPCR) methods to reconstruct sedimentary *Daphnia* DNA (Fig. 1b). Furthermore, we examined the potential sources of sedimentary DNA using bulk samples with and without ephippia and organism remains (Fig. 1d) and pore water (Fig. 1e). Potential factors driving sedimentary DNA concentrations were also considered, including enzymatic inhibitors during qPCR (Fig. 1c), and co-sinking with phytoplankton debris and their aggregations (Fig. 1b). Based on the results, we discuss the importance of eDNA released during the growing population and resting stage as a tool for quantifying the abundance of natural *Daphnia* populations.

## Results

### Sedimentary DNA concentration of *Daphnia*

**Figure 2:**
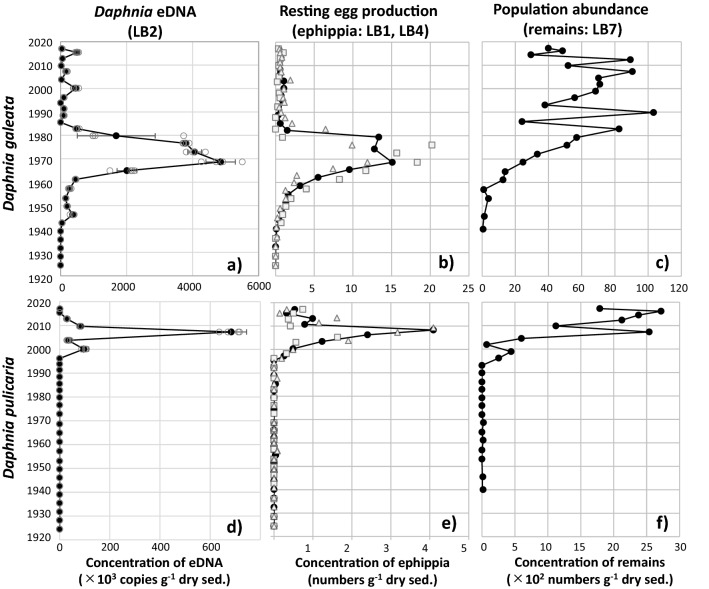
Temporal changes in sedimentary *Daphnia* DNA concentrations (copies of 12S rRNA gene g^−1^ of dry sediments; (**a**,**d**), ephippia (**b**,**e**); and remain (**c**,**f**) abundance (number g^−1^ of dry sediments). Data for *Daphnia galeata* in (**a**)–(**c**); and for *Daphnia pulicaria* in (**d**)–(**f**). For panels (**a**) and (**d**), the open circles indicate raw data, the black closed circles show the average mean (*n* = 4), and the error bar of each data point denotes the SD. For panels (**b**) and (**e**), the grey square and triangles show the data of core LB1 and LB4, respectively, and the black circles indicate the average mean (*n* = 2).

The sedimentary DNA of *D. galeata,* which was analyzed by qPCR analysis, was continuously detected in three out of four replicates after the year 1940 (> 24.5-cm depth; Fig. 2a). Meanwhile, before 1940 (< 24.5-cm depth), sedimentary DNA was not almost detected (Fig. 2a, Supplementary Table S2). The sedimentary DNA of *D. pulicaria* was continuously detected in two out of four replicates after 1994 (0–8.5-cm depth; Fig. 2d). However, before 1994, sedimentary DNA was sporadically detected in one or two replicates from approximately 1988–1980 (10.5–13.5-cm depth) and approximately 1950 (21.5-cm depth; Fig. 2d). The mean DNA copy number in both *Daphnia* species varied significantly depending on the core depth (Supplementary Table S2). Moreover, the total mean DNA copy number of *D. pulicaria* was less than that of *D. galeata* and approximately 4.6% of that of *D. galeata*.

The DNA of *D. galeata* and *D. pulicaria* was not detected in any of the negative controls for sub-sampling, DNA extraction, PCR analysis, or the no-template control (NTC)*,* indicating that sample contamination did not occur during these processes. In addition, through the direct sequencing of PCR amplicons via qPCR assay of *D. galeata* and *D. pulicaria*, we confirmed that only the DNA of these species was amplified.

A spike test was performed to evaluate the effect of PCR inhibition using sediment samples from the LB2 core (Fig. 1c). All the ΔCt values were less than one (ΔCt: − 1.0 to 0.87; Supplementary Table S3), providing no evidence of inhibition.

### *Daphnia* abundance and resting egg production

*Daphnia* remains were sporadically detected depending on the period and species (Fig. 2c, f; Supplementary Table S4). Moreover, the abundance of *Daphnia* ephippium also changed depending on the period (Fig. 2b, e). The abundance of *D. galeata* remains was very low before 1960 and increased significantly thereafter, showing more than 25-fold increase in the late 1980s compared to the early 1950s (Fig. 2c). After the 1980s, the abundance stabilized, although sporadic changes were detected. *D. galeata* ephippia were also rare in the sediment layers dated before 1960 and rapidly increased until the early 1980s (Fig. 2b). However, the ephippia abundance of *D. galeata* significantly decreased after the 1980s. The abundance of *Daphnia pulicaria* remains were extremely limited before the mid-1990s and showed a significant increase of more than 100-fold after that time (Fig. 2f). *D. pulicaria* ephippia were also rare in the sediment layers dated before the mid-1990s and sharply increased until the late 2000s (Fig. 2e). However, similar to *D. galeata*, the ephippia of *D. pulicaria* showed a significant decrease from approximately 2010, although their abundance has remained consistent since that time.

### Relationships between sedimentary *Daphnia* DNA and ephippia, remain concentrations

**Figure 3:**
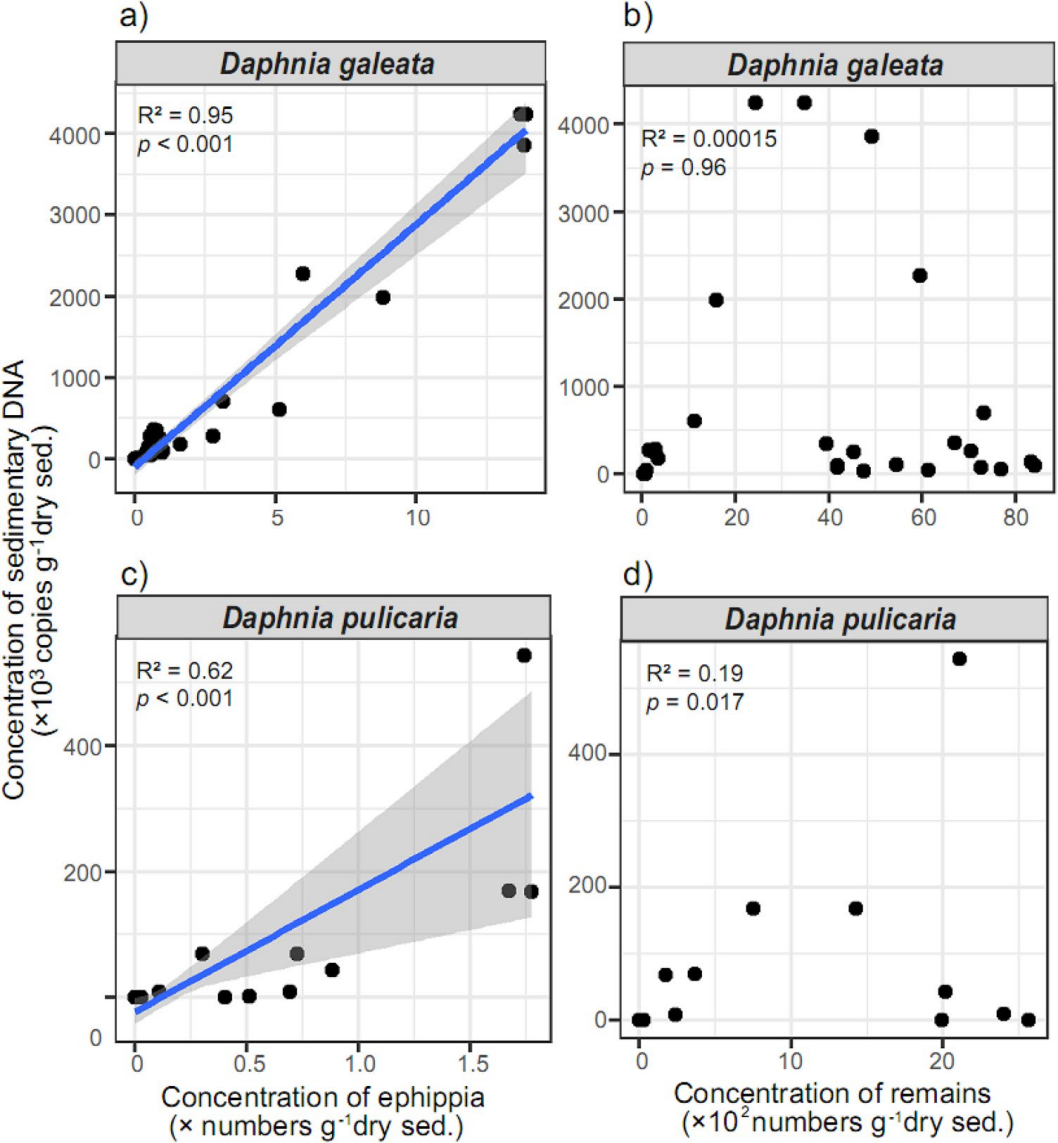
Relationships among the sedimentary DNA (copies of 12S rRNA gene g^−1^ of dry sediments), ephippia, and remain abundance (number g^−1^ of dry sediments) of *Daphnia*. The blue line denotes a regression line of Gaussian type II regression model with a 95% confidence interval indicated by the gray zone.

A significant correlation was detected between the sedimentary DNA concentration for *D. galeata* and its ephippial concentration during the past 100 years (1920–2020) (type II regression model: *R*^2^ = 0.95, *P* < 0.001, and n = 29; Fig. 3a). The significant correlation was also detected for *D. pulicaria* between the sedimentary DNA and its ephippial concentration (*R*^2^ = 0.62, *P* < 0.001, and n = 29; Fig. 3c), although the correlation is stronger with *D. galeata* than *D. pulicaria*. Conversely, no significant relationship was observed for *D. galeata* between the sedimentary DNA concentration and the remain abundance (*R*^2^ = 0.00015, *P* = 0.96, n = 29; Fig. 3b). Whereas a weak relationship was detected for *D. pulicaria* between the sedimentary DNA concentration and its remains (*R*^2^ = 0.19, *P* = 0.017, n = 29; Fig. 3d), but their rapid decline of *D. pulicaria* DNA concentration in the time series after 2010 (Fig. 2d) greatly differed from the abundance of remains (Fig. 2f). However, the decline of DNA concentration of *D. pulicaria* was very similar to the trend observed for the ephippia (Fig. 2e).

### Potential source of *Daphnia* DNA archived in sediments

*Daphnia* eDNA was not detected in the pore water of the three samples from LB2 (Fig. 1e) by qPCR assays (Table 1). However, *D. galeata* DNA was detected in two (LB2-15 and LB2-30) of the three residual sediment samples, while *D. pulicaria* DNA was detected in one (LB2-30). To investigate sedimentary DNA sources, we considered both sieved and non-sieved samples (Fig. 1d). The sieved samples excluded the remains and ephippia, whereas the non-sieved samples were likely to include remains and ephippia. *Daphnia* DNA was detected in both sieved and non-sieved samples (Table 2), except for *D. pulicaria* in the LB2-17 sample whose estimated age was approximately 1968 when the species was thought not to exist in this lake. High concentrations of sedimentary DNA for both *D*. *galeata* and *D. pulicaria* were detected in the non-sieved samples with concentration averaging 865,334 and 938,788 copies g^−1^, respectively.

**Table 1:** 12S rRNA gene copy numbers (copies g^−1^ of dry sediment) for each species for pore water in sediment and its residual pore water-free sediment samples.

Sample ID (estimated age variation in sample range and its age error; yr)	Core depth		Pore water in sediment sample (copies mL^−1^)	SD	Detection/total replicates	Pore water-free sediment sample (mean copies g^−1^ dry)	SD	Detection/total replicates
	Top (cm)	Bottom (cm)						
***Daphnia galeata***								
LB2 1-5 (2007–2017 ± 0–0.4)	0	5	ND	–	0/4	ND	–	0/4
LB2 10-15 (1977–1988 ± 1.7–4.0)	10	15	ND	–	0/4	994,088	176,791	4/4
LB2 25-30 (before 1924–1935 ± over 29)	25	30	ND	–	0/4	812	827	2/4
***Daphnia pulicaria***								
LB2 1-5 (2007–2017 ± 0–0.4)	0	5	ND	–	0/4	ND	–	0/4
LB2 10-15 (1977–1988 ± 1.7–4.0)	10	15	ND	–	0/4	ND	–	0/4
LB2 25-30 (before 1924–1935 ± over 29)	25	30	ND	–	0/4	210	212	2/4

**Table 2:** 12S rRNA gene copy numbers (copies g^−1^ of dry sediment) for *Daphnia galeata* and *Daphnia pulicaria* in sediment samples with and without sieving.

Sample ID (estimated age in sample depth and its age error; yr)	Core depth		Sample with sieve (mean copies g^−1^ dry)	SD	Detection/total replicates	Sample without sieve (mean copies g^−1^ dry)	SD	Detection/total replicates
	Top (cm)	Bottom (cm)						
***Daphnia galeata***								
LB2-5 (2007 ± 0.4)	4	5	69,902	46,078	3/4	178,869	98,433	4/4
LB2-7 (2000 ± 0.6)	6	7	248,105	105,623	4/4	310,704	85,888	4/4
LB2-17 (1969 ± 6)	16	17	1,191,619	215,515	4/4	2,106,429	460,192	4/4
***Daphnia pulicaria***								
LB2-5 (2007 ± 0.4)	4	5	938,406	150,537	4/4	1,573,205	445,566	4/4
LB2-7 (2000 ± 0.6)	6	7	295,429	136,255	4/4	304,372	135,772	4/4
LB2-17 (1969 ± 6)	16	17	ND	–	0/4	ND	–	0/4

The sedimentary DNA concentration of *D. galeata* and *D. pulicaria* showed almost no correlation with the other proxies, such as pigment concentrations *a* and algal remains, except for the weak negative correlation between the sedimentary DNA concentration of *D. galeata* and diatom valves (Supplementary Table S7), although both long-term trends were different from each other (Tsugeki et al. in preparation).

### *Daphnia* DNA concentration in ephippia

The sample list used to estimate *Daphnia* DNA concentrations in the ephippia is shown in Supplementary Table S5. We detected DNA from the ephippia with resting eggs of *D. galeata* (66,692 ± 24,775 copies ephippia^−1^). However, no DNA was detected from their ephippia without resting eggs (Supplementary Table S6). Conversely, DNA was detected from the ephippia with and without resting eggs of *Daphnia pulicaria*. It should be noted that their mean DNA concentrations per ephippia with resting eggs (88,038 ± 33,807 copies ephippia^−1^) were significantly higher than those without resting eggs (6592 ± 1881 copies ephippia^−1^). Moreover, for *D. pulicaria* DNA concentration in the ephippia with resting eggs approximately corresponded to the *Daphnia* sedimentary DNA concentration (Supplementary Tables S2 and S6).

## Discussion

This study demonstrated continuously quantitative reconstruction of *Daphnia* eDNA from sediment layers in a 100-year period using high-resolution dating. Although previous studies have reported the long-term preservation of aquatic zooplankton eDNA in the sediments of freshwater^36,37^ and marine systems^38,39^, however continuous and quantitative reconstruction of zooplankton sedimentary DNA has not been previously reported^40,41^. Furthermore, this study has also shown that the historical variation of *Daphnia* eDNA from sediment layers reflect spawning and reproductive activities than population abundance.

One may suspect that several factors, such as diagenetic degradation, drive the temporal variation in the *Daphnia* eDNA concentrations in the sediments. However, the concentrations of sedimentary *Daphnia* DNA were not always higher in the upper layers than in the lower or middle layers, suggesting that the variation in sedimentary DNA concentration cannot be attributed to time-dependent diagenetic degradation. Although leaching from sediments has predominately been observed in coarse-textured or unsaturated deposits that allow fluid advection across strata^42,43^, it is generally regarded as a minor process in permanently water-saturated sediments^44^. Successive compaction is associated with sedimentation over an extended period. Thus, it is possible that upward fluid advection in the surface layer occurs continuously, enhancing DNA leaching. However, this study did not detect *Daphnia* eDNA in any of the pore water samples (Table 1), which is similar to the results of another qPCR study of aquatic sediment core^45^. Therefore, it is reasonable to conclude that leached eDNA from Lake Biwa only negligibly influences sedimentary DNA. Since the sedimentary DNA concentration of *Daphnia* species showed almost no correlation with the other proxies, such as pigment concentrations and algal remains. Thus, sedimentary *Daphnia* DNA seems not to be regulated by co-sinking with phytoplankton debris and/or their aggregations.

In Lake Biwa, temporal variations in sedimentary *Daphnia* DNA concentrations are not consistent with their abundance. However, they are consistent with their resting egg production. In this lake, the variation in the sedimentary DNA concentration of two *Daphnia* species differed between species over several decades. For *D. galeata*, the sedimentary DNA concentration exhibited an increase from the 1960s in parallel with increases in their abundance and resting eggs numbers, indicated by their analyzed remains and ephippia, respectively. Previous microscopic observations of *Daphnia* remains and ephippia also revealed a significant increase in *D. galeata* from the 1960s to the 1970s, when increased eutrophication of the lake^29,34,35^ boosted ephippial abundance due to substantial increases in *Daphnia’s* population density^29^. However, after the 1970s, the sedimentary DNA concentration rapidly decreased in parallel with declining ephippial abundances, despite abundant *Daphnia* remains indicating that this species continued to dominate the zooplankton community in the lake^34,46^. Moreover, we detected a similar trend for *D. pulicaria*. In Lake Biwa, *D. pulicaria* was thought to appear abruptly in the year 1999^47^. In accordance with these findings, we did not detect significant sedimentary DNA concentrations of *D. pulicaria* before 2000. However, after 2000, the abundance and resting egg numbers of *D. pulicaria* significantly increased, resulting in a simultaneous increase in their sedimentary DNA concentrations. The sedimentary DNA and ephippial abundance of *D. pulicaria* rapidly decreased after 2010 even though the species remained abundant in the lake, indicated by the significant remains collected in the study core. In other lakes, discrepancies between the population abundance and resting egg production for *Daphnia* species have also been demonstrated in sedimentary records^48^. Therefore, the discrepancy between the sedimentary DNA concentrations and their abundance of *Daphnia* species in Lake Biwa suggests that sedimentary *Daphnia* DNA is not an effective tool for estimating past population abundance. However, it can be used to reconstruct the temporal variation of eDNA released during the reproductive activities related to resting egg production.

Despite the wide application of eDNA as a monitoring tool, scientists continue to debate the use of eDNA as a quantitative indicator^49^. Several studies have shown that eDNA concentrations in water are positively correlated with the number of individuals and biomass of a species^12,14^. Thus, eDNA could be used an indicator of these aquatic species^12,13^. However, higher eDNA release rates were observed during spawning periods^16–18^. In this study, the observed historical records of sedimentary *Daphnia* DNA were consistent with their variation in resting egg production, not their abundance, suggesting that eDNA released during spawning activities is a significant source of sedimentary *Daphnia* DNA. Therefore, we must consider the possibility that eDNA concentrations in water may not provide an accurate representation of the population numbers or biomass of aquatic species, and that sedimentary DNA could be used as a powerful tracking tool for spawning production of species. The biological source represented by sedimentary DNA concentrations of *Daphnia* remains limited. Several studies have suggested that fish eDNA is derived from the reproductive materials of organisms, such as eggs and sperm^50^. In consistent with previous evidence, we detected high concentrations of sedimentary DNA in both *Daphnia* species from the non-sieved samples likely including ephippia compared to those in the sieved samples excluding ephippia, implying that the material derived from spawning activity raised sedimentary *Daphnia* DNA concentration (Table 2). Besides, extracellular DNA might be also represented as a source of sedimentary DNA^51,52^. These findings suggest that to further understanding of the potential sources in sedimentary *Daphnia* DNA, more research is required to determine the type of eDNA released during spawning or hatching activities and the time at which eDNA is released.

On the other hand, the application of environmental messenger RNA (mRNA) to these investigations may prove useful for determining the DNA sources, because mRNA expression changes depending on the physiological conditions of a species (such as reproduction and dormant). For *Daphnia*, the mRNA-cDNA of the Chk1 gene regulates oocyte maturation during reproduction^53^ and Caspase-3 mRNA increases with aging related to the appearance of sexual reproduction^54^. Recent studies have indicated advances in these techniques by demonstrating that eRNA can be detected from water^55^ and sediment core samples^56^. Furthermore, it has been shown by using sedimentary mRNA that some zooplankton were surviving in dormancy in sediments and possibly use cytochrome c oxidase (COX) to exit dormancy^56^. Therefore, a combined analysis of sedimentary DNA and sedimentary mRNA could possibly achieve a clear and comprehensive reconstruction of the historical variation in reproductive activity and sources of eDNA for the *Daphnia* species in Lake Biwa.

In conclusion, in this study, we continuously and qualitatively detected sedimentary *Daphnia* DNA for a 100-year period, demonstrating the potential of long-term qualitative zooplankton eDNA detection from sediments as shown in other aquatic organisms such as fish^45^ and plankton eDNA^57^. The absence of sedimentary *Daphnia* DNA in the pore water samples indicated that the temporal succession of sedimentary *Daphnia* DNA records was not vertically disturbed. The temporal changes in the concentrations of sedimentary *Daphnia* DNA were very similar to their ephippia abundance, showing the resting egg production differed from their population density. Therefore, sedimentary *Daphnia* DNA is a possible indicator of the magnitude of the resting reproductive activities of *Daphnia* in water. Overall, we confirmed that sedimentary *Daphnia* DNA can be used as a proxy for monitoring the spawning activity of the species, and the proxy can be tracked in 100-year sedimentary records.

## Methods

### Sampling site and sediment collection

Paleolimnological analysis by using sediment core samples were applied to reconstruct historical variations of *Daphnia* eDNA concentrations in Lake Biwa (Fig. 1b). Lake Biwa is the largest monomictic and mesotrophic lake in Japan. In this lake, during the last several decades the industrial revolution, multiple stressors of human origins impacted this ecosystem and the resident biological communities^34,35,58^. In our study, four 30-cm-long gravity core samples (namely LB1, LB2, LB4, and LB7; Fig. 1a–e) were collected on 17 August 2017 at the anchoring site of *Hasu*, a Center of Ecological Research boat from Kyoto University (Supplementary Fig. S2). A gravity corer with an inner diameter of 10.9 cm and a length of 30 cm was used to obtain the core samples. LB7 core was analyzed for chronological and reconstruction of temporal variation in *Daphnia* remain abundance (Fig. 1a,b). LB2 and LB1, LB4 cores were analyzed for reconstruction of temporal variation in sedimentary *Daphnia* DNA concentrations and resting egg production, respectively (Fig. 1b). Additionally, two 30-cm-long gravity core samples (namely IM1 and IM8; Fig. 1c,f) were collected at a pelagic site in the northern basin of Lake Biwa in August 2019 (Supplementary Fig. S2). The collected cores were sectioned at intervals of 1-cm thickness using a vertical extruder with a cutting apparatus, except for core number IM8, which was sectioned at 5-cm intervals (Fig. 1f). During the sectioning process, several millimeters of outer edge in each layer disturbed during the splitting process were carefully removed from the entire samples using a knife. After sectioning, each sliced sample were homogenized by shaking and then, all subsamples were taken from each homogenized sample. The pipes, knives, and cutting apparatus were cleaned with 0.6% sodium hypochlorite, tap water, and Milli-Q water to avoid DNA cross-contamination. Each sliced sample was transferred to lightproof bags and frozen at − 80 °C until further analysis.

To examine the contamination due to core splitting, DNA extraction, and qPCR analysis, control water samples were inserted at a depth of 14.5–29.5 cm in the sediment cores, and the water samples for core IM1 were used as the negative control (Fig. 1c).

### Chronology of sediment cores

Sediment chronology was performed for the LB7 core based on the constant rate of supply (CRS) method of ^210^Pb dating^59^ and verified using the ^137^Cs peak traced in the period 1962 to 1963^60^. Details of the chronological method have been reported elsewhere^61^. Briefly, dried samples were sealed in holders for a month to allow ^222^Rn and its short-lived decay product (^214^Pb) to equilibrate, which were determined by gamma counting using a germanium detector (GXM25P; EG & G ORTEC, Tokyo, Japan) equipped with a multi-channel analyzer (MCA7700; SEIKO EG & G, Tokyo, Japan) at the Center for Marine Environmental Studies, Ehime University. The activity of supported ^210^Pb was estimated by measuring the activity of ^214^Pb, whereas that of ^210^Pb_excess_ was determined according to the difference between the total and the supported ^210^Pb (^210^Pb_excess_ = ^210^Pb_total _− ^214^Pb). The age and age error of the remaining cores (LB1, LB2, and LB4) were indirectly estimated using stratigraphic correlations between the cores based on chronological controls in chlorophyll pigments and magnetic susceptibilities of the chronological LB7 core^61^. To compare these proxies, the marked peak or trough layers were used as reference layers (Supplementary Fig. S3).

### DNA extraction and purification

DNA extraction in the sediment samples was performed according to methods described in previous studies^45,62^. In brief, 9 g of each sediment sample was incubated at 94 °C for 50 min in a 9 mL alkaline solution comprising 6 mL of 0.33 M sodium hydroxide and a 3 mL Tris–EDTA buffer (pH 6.7). After centrifugation at 10,000×*g* for 60 min, 7.5 mL of the supernatant of the alkalized mixture was neutralized with 7.5 mL of 1 M Tris–HCl (pH 6.7). After adding 1.5 mL of 3 M sodium acetate (pH 5.2) and 30 mL absolute ethanol, the solution was preserved at − 20 °C for more than 1 h and then centrifuged at 10,000×*g* for 60 min. The pellet was transferred into a power bead tube that was installed in a fecal-soil DNA extraction kit (Power Soil DNA Isolation Kit, Qiagen, Germany). The ‘Experienced User Protocol 3 to 22’ of the Power Soil DNA Isolation Kit was followed. Finally, 200 μL of the DNA solution was obtained and stored at − 20 °C until qPCR analysis.

### 12S rRNA gene primer-prove development for *Daphnia geleata* and *Daphnia pulicaria*

As the primer–probe for *Daphnia galeata* and *D. pulicaria* in qPCR analysis were not purchased by a company, thus we developed them for the two *Daphnia* species (see Supplementary Table S1). We preliminary obtained the mitochondrial 12S, 16S and COI gene of *Daphnia* genus from the National Center for Biotechnology Information (NCBI, http://www.ncbi.nlm.nih.gov/) and compared among them. From the preliminary results, we decided to use 12S because of the variability of sequences among *Daphnia* genus. Then we obtained the 12S sequences of *Daphnia* genus and other inhabiting plankton species in Lake Biwa, including Copepoda. We designed the primer–probe using Primer3plus (https://www.bioinformatics.nl/cgi-bin/primer3plus/primer3plus.cgi). The reference sequences for the targeted gene regions are queried for potential amplicons between 50–150 bp using NCBI primer blast. The specificities of the primers and probes were then assessed in silico with homologous sequences from other *Daphnia* species in Japan using NCBI targeting 154 bp of the mitochondrial 12S rRNA gene. Once suitable amplicons are found the respective primers and probes are tested against template DNA originating from the species of *D. galeata and D. pulicaria* to verify amplification. During the in silico screening for specificity, we performed Primer-BLAST (http://www.ncbi.nlm.nih.gov/tools/primer-blast/). We checked all species from Japan of the order *Daphnia*. Using the *D. galeata* primer-set, we did not detect any *Daphnia* species. However, the *D. pulicaria* primer can amplify *D. pulex* DNA*,* as these species are known to have very similar sequences^47^. In Lake Biwa, another subgenus *Daphnia* (*D. pulex* group) different from *D. pulicaria* was temporally found around during the 1920s, although thereafter it was never reported^47^. Thus, the *D. pulicaria *primer may temporally detect another subgenus *Daphnia* (*D. pulex group*). However, their appearance time do not overlap, therefore we used the primer for our measurement to detect *D. pulicaria* during the last several decades.

### Quantitative PCR

The DNA samples were quantified by real-time TaqMan quantitative PCR using the PikoReal Real-Time PCR system (Thermo Fisher Scientific, Waltham, MA, USA). The primer–probe sets for the two *Daphnia* species were used for qPCR (Supplementary Table S1). The TaqMan reaction contained 900 nM of each forward and reverse primer, 125 nM TaqMan-Probe, 5 μL qPCR master mix (TaqPath; Thermo Fisher Scientific), and 2.0 μL sedimentary DNA solution. The final volume of the PCR was 10 μL after adding distilled water (DW). The qPCR conditions were as follows: 50 °C for 2 min and 95 °C for 10 min, followed by 50 cycles of 95 °C for 15 s and 60 °C for 60 s. We used a dilution series of 10,000, 1000, 100, and 10 copies per PCR reaction (n = 4) for the standard curve using the target DNA cloned into a plasmid. The R^2^ values of the standard curves ranged from 0.988 to 0.996 (PCR efficiencies = 93.1–102.0%). The quantitative data of the DNA copies (copies g^−1^ dry sed.) were reported by mean values ± standard deviation, which were calculated from DNA copies µL^−1^ PCR reaction with four replicates including zero (i.e., no detection). We also performed four replicates for each sample and an NTC (n = 4). No positives were detected from the NTC and the negative control of DNA extraction, confirming that there was no cross-contamination in any of the DNA measurements.

To confirm primer specificity, an in vivo test for the primer/probe set was performed using the extracted DNA (10 pg per PCR reaction, n = 4) of *D. galeata* and *D. pulicaria*. In addition, qPCR amplicons were sequenced directly from a positive PCR from each site (n = 21) after treatment with ExoSAP-IT (USB Corporation, Cleveland, OH, USA). Sequences were determined using a commercial sequencing service (Eurofins Genomics, Tokyo, Japan).

### Inhibitor test

Spike tests were performed for the LB2 core sample to evaluate the PCR inhibition effect of several substances and minerals in the sediment samples (Fig. 1c). For the spike test, 1 µL plasmid, including the internal positive control (IPC, 207-bp, Nippon Gene Co. Ltd., Tokyo, Japan;100 copies per PCR reaction), was added to the PCR template with 1.6 µL DNA-free DW. We used the primer and probe sets for IPC as follows:

- IPC1-5′: CCGAGCTTACAAGGCAGGTT
- IPC1-3′: TGGCTCGTACACCAGCATACTAG
- IPC1-Taq: [FAM] TAGCTTCAAGCATCTGGCTGTCGGC [TAMRA].

To measure the relative degree of PCR inhibition in the samples, the Ct shift was compared between the samples and controls with the same number of known target DNA copies. The presence of PCR inhibitors was evaluated as ΔCt = Ct sample − Ct positive control. ΔCt ≥ 3 cycles was considered evidence of inhibition^63^ because the presence of PCR inhibitors will delay the Ct with a given quantity of template DNA.

### *Daphnia* abundance and resting egg production as potential sources of *Daphnia* DNA archived in sediments

To unveil the potential source of sedimentary DNA of *Daphnia*, we reconstructed the historical variation in *Daphnia* abundance by counting remains of the post abdominal claw for LB7 core. There are two dominant *Daphnia* species: *D. galeata* Sars (*Hyalodaphnia*) and *D. pulicaria* Forbes (*Daphnia*)^47,61^, which have different post-abdominal claw characteristics^64^ and are known to be preserved in centuries-old sediments^65^. The post-abdominal claw remains were counted for core LB7 from the surface to a depth layer of 21.5 cm and additionally 23.5 cm, 25.5 cm, and 29.5 cm, totaling 25 samples, though each layer was expressed as mid-depth; e.g., 0.5 cm for the 0–1 cm depth layer. The enumeration method was based on a simplified standard method^65^ as previously reported^29^.

*Daphnia* resting eggs enveloped by thickened carapaces, referred to as ephippial cases, and these ephippia can be preserved in sediments for decades to centuries^29,30,33^. In Lake Biwa, *Daphnia* species in Lake Biwa are distinguished on the basis of the size of the ephippium, with a boundary length of approximately 860 μm between them^61^. We collected ephippia from the surface to a depth layer of 29.5 cm for cores LB1 and LB4 (except for several layers of the LB1 core), totaling 56 samples (Supplementary Table S4). A detailed method for collecting ephippia is described in a previous study^61^. The total number of collected ephippia with an almost perfect shape, namely complete formation, or with a partial body constituting more than half of the original shape, namely incomplete formation, are shown in Supplementary Table S4. In our study, at least 16 ephippia in each sample were measured by photographs taken by a digital camera, excluding those from the samples in which fewer than 16 complete ephippia were detected (Supplementary Fig. S4). Species identification was then performed based on length.

To determine whether the *Daphnia* sedimentary DNA concentrations were regulated by DNA derived directly from *Daphnia* remains or ephippia included in the analytical sediment, we divided the sediment sample into two fractions to exclude the remains and ephippia (Fig. 1d). The minimum size of *Daphnia* remains in this lake was approximately 55 μm (Tsugeki et al., in preparation). The analytical sediments for DNA extraction were divided into particles < 38 μm and > 38 μm using 38-μm mesh sieves on three-layer samples (specifically, LB2-5; 4.5 cm, LB2-7; 6.5 cm, and LB2-17; 16.5 cm expressed in middle depth of each sample) for core sample LB2, whose layers were known to include abundant ephippia and *Daphnia* remains. Furthermore, to test the possibility of the vertical movement of *Daphnia* sedimentary DNA through pore waters, we examined the sedimentary DNA concentration in pore water and its residual sediment by qPCR analysis (Fig. 1e). All DNA extractions were evaluated for sediment with and without sieves, and pore waters and the associated residual sediment samples were evaluated according to previous studies^45^.

### Measurement of DNA concentration in sediment ephippia

To determine the potential source of sedimentary *Daphnia* DNA, we quantified the DNA concentration extracted from several ephippia obtained from the 0–5 cm and 5–10 cm layers of core IM8 using qPCR analysis (Fig. 1f). We selected 34 and 23 ephippia for *D. galeata* and *D. pulicaria*, respectively. We then measured the ephippial lengths and determined whether they contained resting eggs using a microscope. Among the selected ephippia, the well-preserved 17 ephippia with almost complete formation were set aside and grouped into 6 samples together in two or three ephippia for DNA analysis (Supplementary Table S5). Grouping was performed because of the low DNA concentrations typically associated with individual ephippium^61^.

### Possible factors regulating sedimentary *Daphnia* DNA

To explore potential factors regulating temporal variation in sedimentary DNA concentrations, we analyzed chlorophyll pigments and algal remains. Sedimentary pigments of chlorophyll *a* were investigated for the LB 2 core, and algal remains were investigated for the LB7 core (Fig. 1a). Details of the method used for chlorophyll-*a* and algal remains are described in previous study^61^. In short, the concentrations of chlorophyll-*a* and phaeopigments were calculated according to the method^66^ and the diatom remains were analyzed according to the simplified method^67^. Green algae, *Micrasterias hardyi, Staurastrum dorsidentiferum*, *S. arctiscon*, *S. limneticum*, *S. pingue, *and* Pediastrum biwae*, were enumerated in a Sedgewick–Rafter chamber, following the method of zooplankton enumeration.

### Data analysis

Regression models along with the standardized major axis method were used to determine the relationship between the sedimentary DNA concentration obtained from qPCR analysis and abundance or resting egg production in the sediment layers. Since qPCR (LB2), remains (LB7), and ephippia (LB1, LB4) analyses were performed on different cores, the chronological age of each analytical sample differed slightly. Therefore, prior to performing the statistical analysis, the sedimentary DNA (LB2) and ephippia data (LB1, LB4) in each chronological age were converted to annual data by linear interpolation and averaged for the year corresponding to the period in each sample of the chronology core (LB7). This conversion was possible because the time resolution at 1-cm intervals represented several years, depending on the sediment depth^29,61^. We employed the Gaussian type II model because our preliminary evaluation showed higher R^2^ values for type II regression models with a Gaussian distribution than for those with a logarithmic distribution, in all cases. All statistical analyses were performed using R ver. 4.0.3 (R Core Team 2020) with the package “smatr” ver. 3.4-8 for type II regressions. The significant criteria of all analyses were set as α = 0.05. In addition, to explore the potential environmental factors driving temporal variation in sedimentary DNA concentrations, we performed Pearson's correlation analysis among the sedimentary DNA concentrations, chlorophyll *a* concentration, and algal remains using the SPSS version 20.0 statistical package.

## Supplementary Information

Supplementary Information.

## Data Availability

Source data are provided with this paper.
