# Supplementary material for: Reconstruction of 100-year dynamics in Daphnia spawning activity revealed by sedimentary DNA
Source: Sci Rep. 2022 Feb 2;12:1741. doi: 10.1038/s41598-021-03899-0 (PMC8810866; doi:10.1038/s41598-021-03899-0)
Supplement: Supplementary file 1 — Supplementary Information. [file 41598_2021_3899_MOESM1_ESM.docx]

**Supplementary Information for Tsugeki et al., titled ‘Reconstruction of 100-year dynamics in *Daphnia* spawning activity revealed by sedimentary DNA.’**

**Supplementary Figures**

Supplementary Figure S1. Schematic diagram of *Daphnia* life cycle with possible sources of sedimentary *Daphnia* DNA and their respective contributions to sedimentary DNA. During favorable conditions, subitaneous reproduction takes place and female *Daphnia* molt frequently and produce offspring. However, during stressful environmental conditions, *Daphnia* switch to resting egg (ephippial) reproduction, that is, long-lived dormant eggs, which preserve in bottom sediment for centuries. The arrow from the claw remains and ephippia with or without resting eggs are transported to the lake bottom and deposited in sediment. This study determined eggs in ephippia for resting state to be one of the main sources of sedimentary *Daphnia* DNA.


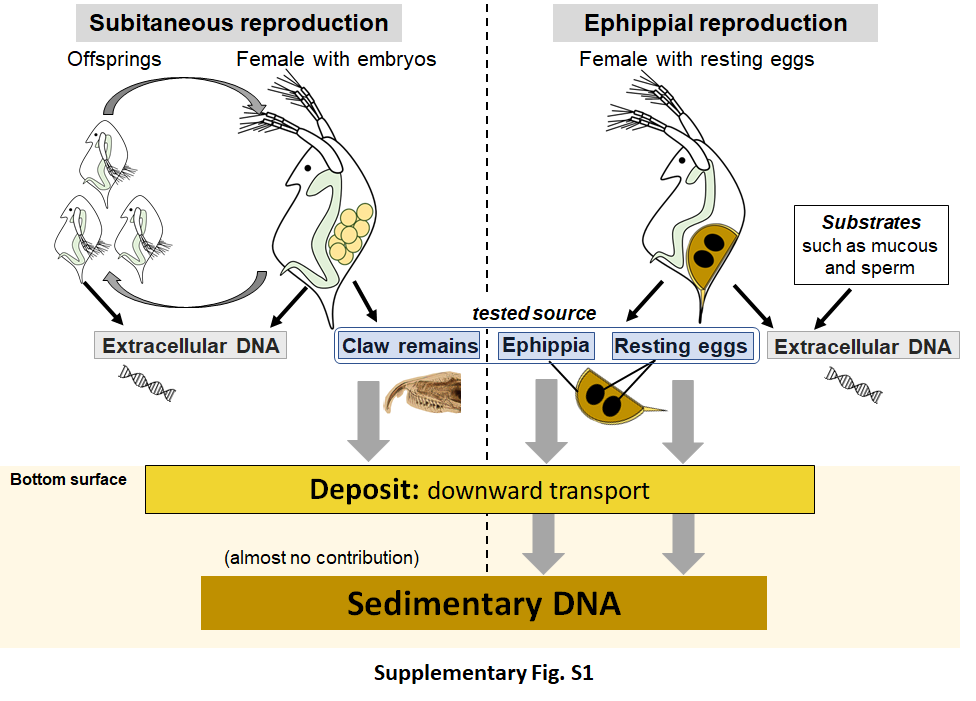


Supplementary Figure S2. Map of sampling area and coring site in Lake Biwa, Japan.

Supplementary Figure S3. Profiles of (a) the chlorophyll-a pigments including its derivatives and (b) magnetic susceptibilities in the four cores (LB1, LB2, LB4, and LB7) collected from Lake Biwa. To estimate the chronology of LB1, LB2, and LB4 based on the chronological LB7 core, we performed a comparison of proxies. Three depths were used as reference layers for the pigment proxies, which increased at a depth of 21.5 cm in core LB7 (estimated date: 1953.1 ± 12.1), peaked at a depth of 13.5 cm (1982.8 ± 2.5), and troughed at a depth of 5.5 cm (2007.3 ± 0.4). Similarly, two depths were used as the reference layers for magnetic susceptibility determination: 17.5 cm in core LB7 (estimated date: 1968.6 ± 5.9), which displayed a decreasing trend, and 9.5 cm (1996.2 ± 0.70) that indicated the end of the trend. The reference layers in the three cores are connected by dotted lines.


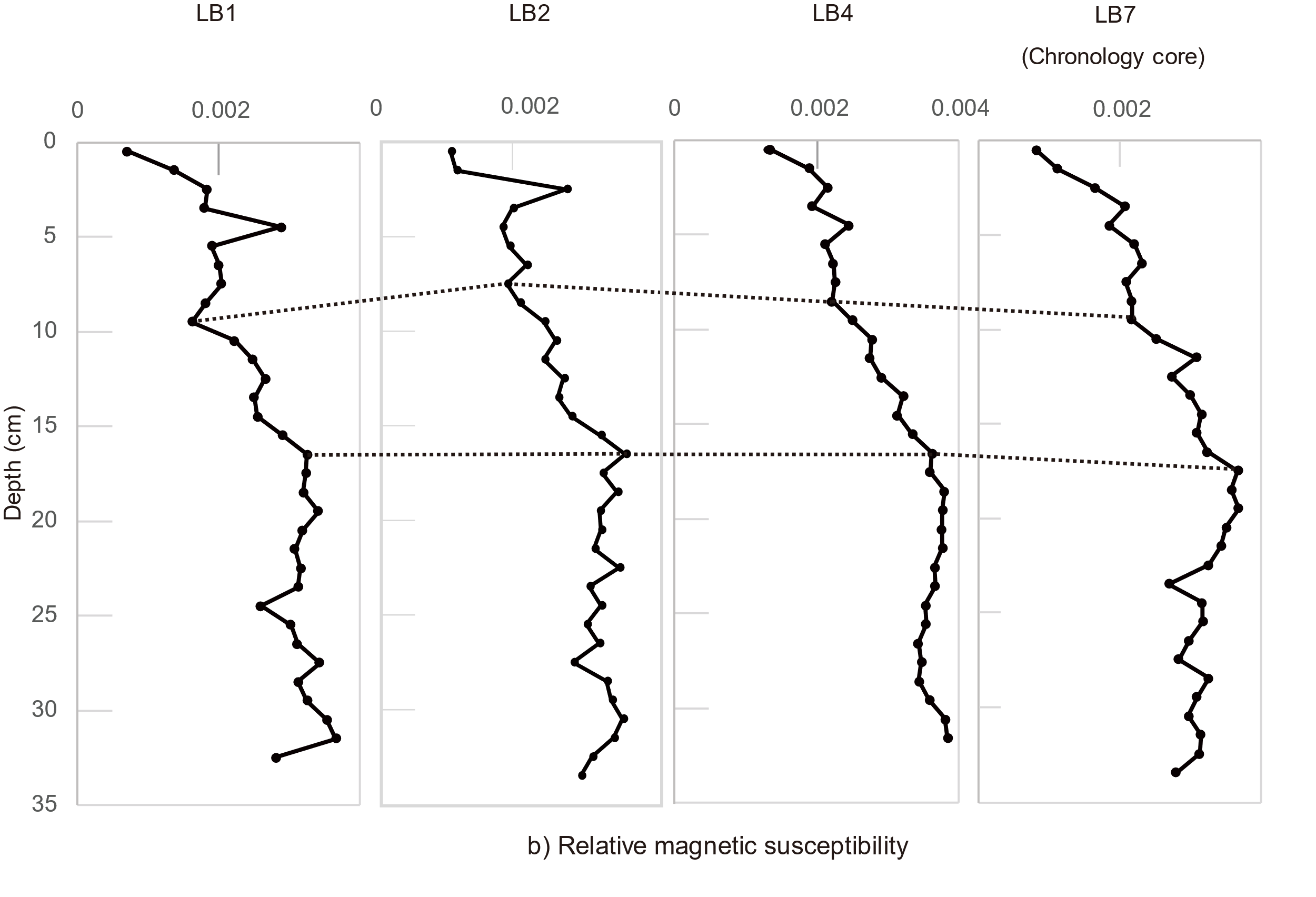

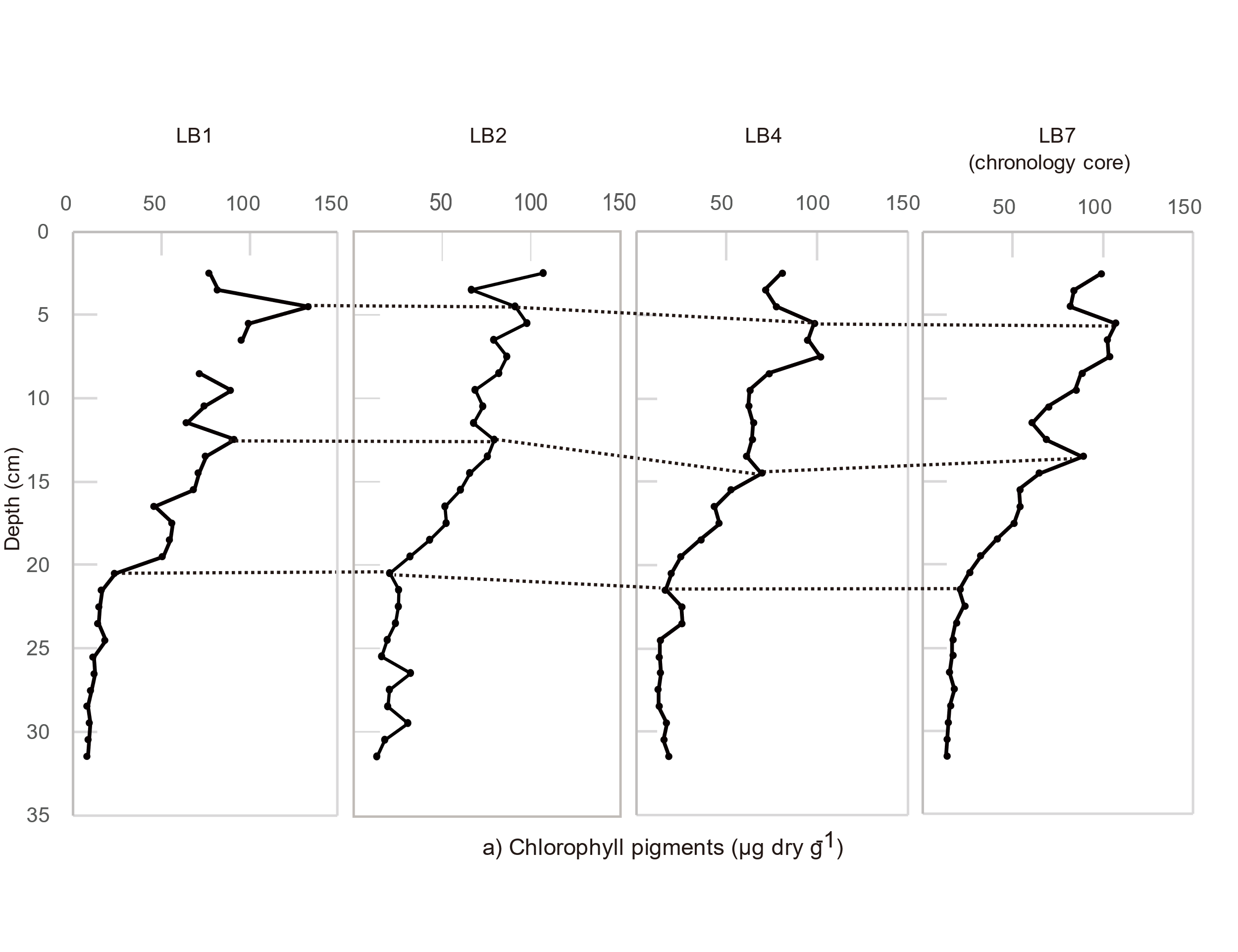


Supplementary Figure S4. Photos of ephippia with (a, c) and without (b, d) resting eggs for *Daphnia galeata* (a, b) and *Daphnia pulicaria* (c, d), respectively, indicating the ephippial length measured in this study.


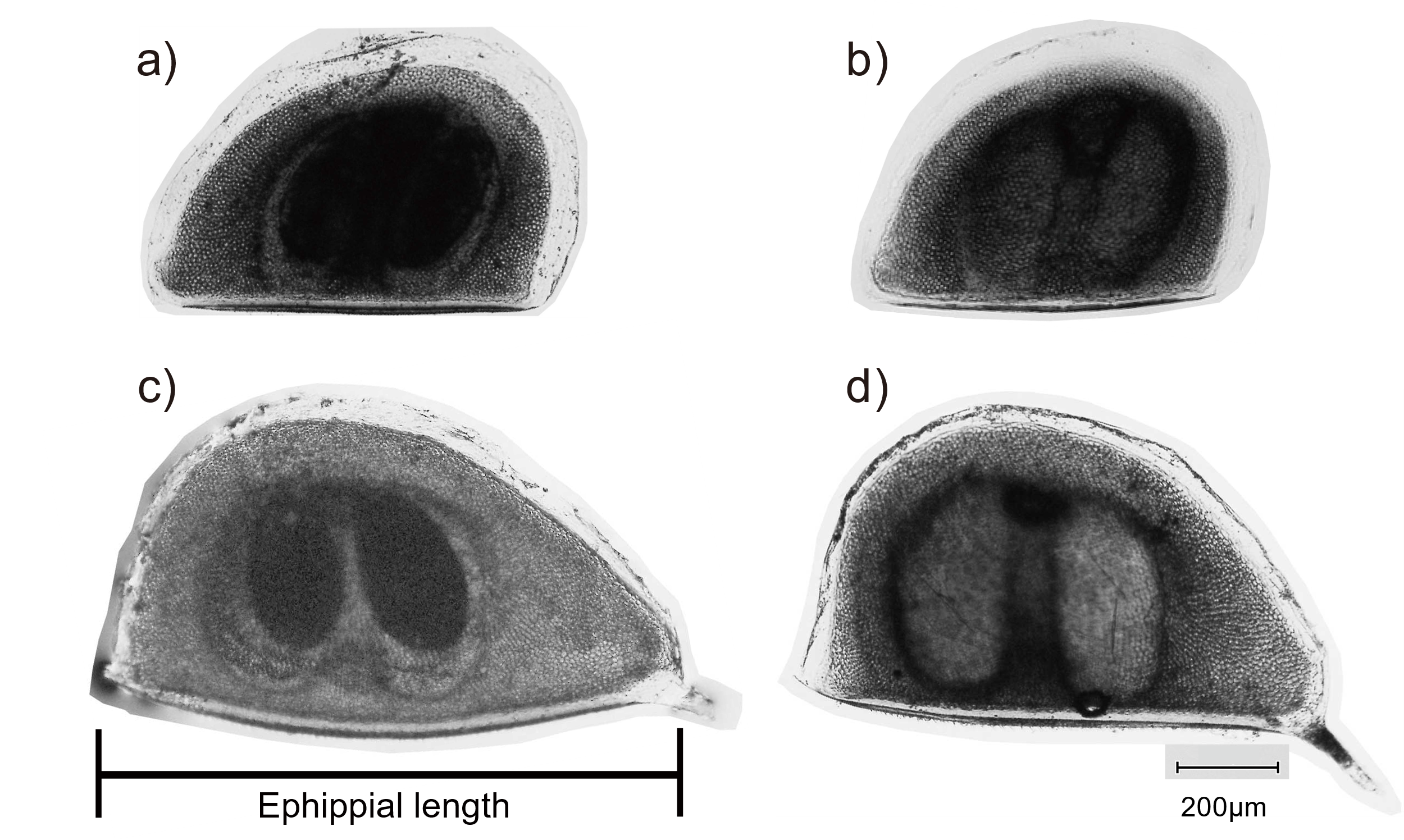


# Supplementary Tables

Supplementary Table S1. Specific primers and probes for the *Daphnia* species targeting 154 base pairs of the mitochondrial 12S rRNA gene.

| **Primer and probe** | **Sequences (5'-3')** | **Sequences (5'-3')** |
| --- | --- | --- |
| **Species** | ***Daphnia galeata*** | ***Daphnia pulicaria*** |
| **Forward primer** | **GTC CCT TAA TCG ATA GTC CTC G** | **CTT GTC CTA TAA TTG ATA GTC CCC A** |
| **Reverse primer** | **TCA CCT CCA CCC CCA TAT AA** | **GTA GCT CAT CAC TGC CCT TTT ATA** |
| **Probe** | **Probe, FAM-AAC TTC AGG TCA AGG TGC AG-TAMRA** | **Probe, FAM-TAC TTC AGG TCA AGG TGC AG-TAMRA** |

Supplementary Table S2. Sample numbers with age, depths, and 12S rRNA gene copy numbers (copies g^-1^ of dry sediment) for the *Daphnia* species collected in this study and their mean with 1SD.

Supplementary Table S3. Results (ΔCt) of the spike test for evaluating the PCR inhibition effect. A ΔCt value < 3 indicated no effect of enzymatic inhibition on PCR amplification for the analyzed sample.

Supplementary Table S4. Results of the remains analysis (LB7) and ephippial numbers (LB1 and LB4) of each *Daphnia* species. The total number of ephippium with an almost complete shape which we collected throughout the core is 1461 and 1131 from LB1 and LB4, respectively. Whereas those with a partial body constituting more than half of the original shape, which expresses incomplete formation, for cores LB1 and LB4 contained 217 and 294 ephippia, respectively.

Supplementary Table S5. The size of two or three ephippia for each sample. The ephippia in shadow area are picked up from 0–5 cm layers, those in others area are picked up from 6–10 cm layers from IM8 core and asterisk (*) means ephippia with resting eggs.

| Species | ***Daphnia galeata*** | | | ***Daphnia pulicaria*** | | |
| --- | --- | --- | --- | --- | --- | --- |
|  | Length of ephippia | | | Length of ephippia | | |
| Sample ID. | (um) | | | (um) | | |
| 1 | 600* | 556* | - | 1000* | 1089* | - |
| 2 | 755 | 622 | 578 | 1044 | 889 | 866 |
| 3 | 600 | 667 | 822 | 866 | 933 | 1000 |
| 4 | 600 | 600 | 578 | 1089 | 933 | 978 |
| 5 | 733 | 622 | 689 | 867 | 889 | 889 |
| 6 | 600 | 711 | 667 | 1044 | 933 | 911 |
|  |  |  |  |  |  |  |
|  | | | | | | |

Supplementary Table S6. 12S rRNA gene copy numbers (copies ephippia^-1^) of one ephippia for *Daphnia galeata* and *Daphnia pulicaria* using the samples shown in Supplementary Table S5.

Supplementary Table S7. Pearson correlation analysis between the concentration of sedimentary *Daphnia* DNA and chlorophyll a, diatoms, green algal remains.
